# *Pyrus betulaefolia* ERF3 interacts with HsfC1a to coordinately regulate aquaporin *PIP1;4* and *NCED4* for drought tolerance

**DOI:** 10.1093/hr/uhae090

**Published:** 2024-03-30

**Authors:** Feng Zhang, Zhijian Pan, Chenyang Han, Huizhen Dong, Likun Lin, Qinghai Qiao, Keke Zhao, Juyou Wu, Shutian Tao, Shaoling Zhang, Xiaosan Huang

**Affiliations:** College of Horticulture, State Key Laboratory of Crop Genetics and Germplasm Enhancement and Utilization, Nanjing Agricultural University, No.1 Weigang, Tongwei Road, Nanjing 210095, China; College of Horticulture, State Key Laboratory of Crop Genetics and Germplasm Enhancement and Utilization, Nanjing Agricultural University, No.1 Weigang, Tongwei Road, Nanjing 210095, China; College of Horticulture, State Key Laboratory of Crop Genetics and Germplasm Enhancement and Utilization, Nanjing Agricultural University, No.1 Weigang, Tongwei Road, Nanjing 210095, China; College of Horticulture, State Key Laboratory of Crop Genetics and Germplasm Enhancement and Utilization, Nanjing Agricultural University, No.1 Weigang, Tongwei Road, Nanjing 210095, China; College of Horticulture, State Key Laboratory of Crop Genetics and Germplasm Enhancement and Utilization, Nanjing Agricultural University, No.1 Weigang, Tongwei Road, Nanjing 210095, China; College of Horticulture, State Key Laboratory of Crop Genetics and Germplasm Enhancement and Utilization, Nanjing Agricultural University, No.1 Weigang, Tongwei Road, Nanjing 210095, China; College of Horticulture, State Key Laboratory of Crop Genetics and Germplasm Enhancement and Utilization, Nanjing Agricultural University, No.1 Weigang, Tongwei Road, Nanjing 210095, China; College of Horticulture, State Key Laboratory of Crop Genetics and Germplasm Enhancement and Utilization, Nanjing Agricultural University, No.1 Weigang, Tongwei Road, Nanjing 210095, China; College of Horticulture, State Key Laboratory of Crop Genetics and Germplasm Enhancement and Utilization, Nanjing Agricultural University, No.1 Weigang, Tongwei Road, Nanjing 210095, China; College of Horticulture, State Key Laboratory of Crop Genetics and Germplasm Enhancement and Utilization, Nanjing Agricultural University, No.1 Weigang, Tongwei Road, Nanjing 210095, China; College of Horticulture, State Key Laboratory of Crop Genetics and Germplasm Enhancement and Utilization, Nanjing Agricultural University, No.1 Weigang, Tongwei Road, Nanjing 210095, China

## Abstract

Environmental disasters like drought reduce agricultural output and plant growth. Redox management significantly affects plant stress responses. An earlier study found that PbPIP1;4 transports H_2_O_2_ and promotes H_2_O_2_ downstream cascade signaling to restore redox equilibrium. However, this regulatory mechanism requires additional investigation. In this search, the AP2 domain-containing transcription factor was isolated by screening Y1H from the wild pear (*Pyrus betulaefolia*) cDNA library, named PbERF3. The overexpression of PbERF3 in pear callus and *Arabidopsis* enhanced plant resistance to drought and re-established redox balance. The transcripts of the *NCEDs* gene were upregulated under drought stress. The drought stress-related abscisic acid (ABA) signaling pathway modulates PbERF3. PbERF3 silencing lowered drought tolerance. Furthermore, yeast 2-hybrid, luciferase, bimolecular fluorescence complementation, and co-immunoprecipitation assays verified that PbERF3 physically interacted with PbHsfC1a. The PbERF3-PbHsfC1a heterodimer coordinately bound to *PbPIP1;4* and *PbNCED4* promoter, therefore activating both the H_2_O_2_ and the ABA signaling pathway. This work revealed a novel PbERF3-PbHsfC1a-*PbNCED4*-*PbPIP1;4* regulatory module, in which PbERF3 interacts with PbHsfC1a to trigger the expression of target genes. This module establishes an interaction between the H_2_O_2_ signaling component *PbPIP1;4* and the ABA pathways component *PbNCED4*, enabling a response to drought.

## Introduction

Changes in the water potential of the environment can exacerbate drought stress. This stress may cause death or disrupt normal biological processes in plants. Drought conditions can cause osmotic stress, which significantly inhibits plant development and agricultural productivity [1]. Plants are subjected to secondary stressors during droughts, including oxidative stress caused by an excess of reactive oxygen species (ROS) [2]. In addition to using ROS as a way to avoid toxicity, plants also use them as signaling mediators to initiate defensive responses [3].

Hydrogen peroxide (H_2_O_2_) forms a portion of the ROS generated by several metabolic activities in cells [4]. ROS induces cellular damage through several mechanisms, such as lipid peroxidation, nucleic acid degradation, and enzyme inactivation [5]. To maintain a low amount of ROS within the cell, many redox and ROS-scavenging mechanisms are necessary [5]. H_2_O_2_ can behave as a signal molecule, controlling a variety of physiological responses [6]. It is speculated that there is a mechanism for transporting H_2_O_2_ that is involved in the signal transduction pathway. A new study found that some aquaporins in plants function as transporters of H_2_O_2_ [7]. OsPIP1;3 was observed to be elevated in highland rice, which exhibits superior drought resistance compared to lowland rice. In contrast, lowland rice did not show any change in expression. This indicates that OsPIP1;3 likely plays a role in the difference in drought resistance between the two types of rice [8]. Plant cultivars have been shown to vary in the transcriptional regulation of major intrinsic protein.

To adapt to a range of environmental stimuli, plants have created networks of transcription factors (TFs) and defense-related proteins that control the process of signal transmission. The drought stress-responsive pathway in plants is mediated by several TFs, including heat-shock transcription factors (HSFs), NAM, ATAF1, and CUC2 (NAC), basic leucine zipper (bZIP), and APETALA2/ERF (AP2/ERF) [9–11]. The AP2/ERF protein superfamily consists of three different families: ERF, AP2, and RAV. These families are characterized by the presence of at least one DNA-binding domain, namely, the AP2 domain [12]. ERF family members consist primarily of proteins that possess an AP2 domain and have a significant number of introns in their genomic sequence [13–15] . To date, researchers have examined a variety of biotic and abiotic stresses, including pathogen infection, salt stress, dehydration, hypoxia, and the stress-related hormones ethylene and abscisic acid (ABA) [16]. RAP2.6, e.g., is an ERF/AP2 TF that may directly bind to the promoters of RD29A and COLD-REGULATED 15A (COR15A), which contains GCC or DRE motifs. This in turn increases their expression in response to ABA and drought signals [17]. The TF gene *MtCBF4* has been shown to have a role in the way tree trunks react to abiotic stresses, including drought and ABA [18].

Structural proteins and other transcriptional regulators can combine to form stable complexes with ERF proteins [16]. ERF TF target selectivity is also greatly influenced by interaction partners [19]. This phenomenon was observed in both stress responses and developmental processes. RAP2.3 and TGA4 have the potential to interact, which can either increase or particularly activate genes that have binding sites for both TFs in their promoters [20]. The transcriptional co-repressors TOPLESS (TPL) and TOPLESS-RELATED (TPR) in *Arabidopsis* can interact with EAR and BDR-containing ethylene response factors (ERFs) [21]. Moreover, PpERF3, PpERF24, and PpERF96 can interact with PpMYB114 and jointly regulate the biosynthesis of anthocyanins in pears [22].

Heat-shock transcription factors are crucial elements of the signal transduction system that are susceptible to various stresses [23]. The plant HSF family has higher diversity in terms of quantity, structure, and regulatory mechanisms compared to other eukaryotes, which typically possess one to three HSFs. This might be attributed to the sessile nature of plants and the crucial role that HSFs have played in their ability to tolerate stress throughout plant evolution [24]. The DNA-binding domain located in the N-terminal region of the HSF protein sequence can accurately identify and bind to the heat-shock protein promoter of the downstream target gene [25]. An oligomerization domain is linked to a DNA-binding domain by a chain of 15–80 amino acid residues. Plant HSFs are categorized into three classes, A, B, and C, according to the structural characteristics of the oligomerization domain [25]. The significance of HSFs in plants was the first and most extensively investigated function in abiotic stress. Overexpression (OE) of AtHsfA1b increased transgenic plant water production, resulting in higher drought tolerance [26]. Transcriptional inhibitors of class B (AtHsfB1 and AtHsfB2b) have a negative effect on the regulation of abiotic stress [27]. It has also been reported that HSFA6a, HSFA4, and HSFA9 are crucial in drought responses [28, 29]. While the importance of HSFs in stress responses is widely acknowledged, further investigation is required to gain a comprehensive understanding of the specific role that class C HSFs play in stress tolerance, as well as the various other functions performed by HSFs.

Here, it was found that the PbERF3-PbHsfC1a-*PbNCED4*-*PbPIP1;4* module was essential for the sensing and transduction of redox and osmotic signals in pear plants that are caused by drought stress. The current research demonstrated that PbERF3 enhanced drought resistance in pears by interacting with PbHsfC1a, which binds to the promoters of *PbPIP1;4* and *PbNCED4* to positively regulate the transport of H_2_O_2_ and the biosynthesis of ABA in response to drought tolerance. To precisely adjust cellar redox sensing and transmission in response to drought stress, this study thus reveals a unique regulatory network.

## Results

### Sequence analysis and Y1H-based screening of PbERF3

It has been previously identified that a plasma membrane intrinsic protein PbPIP1;4 is capable of stimulating downstream H_2_O_2_ cascade signaling in response to drought stress *via* H_2_O_2_ translocation [30]. Using a Y1H test to screen a *Pyrus betulaefolia* cDNA library, the molecular processes behind the increased drought tolerance and H_2_O_2_ transport were investigated. After performing sequencing, the protein called ERF and designated as *PbERF3* (Pbr030451.1) was identified.

**Figure 1:**
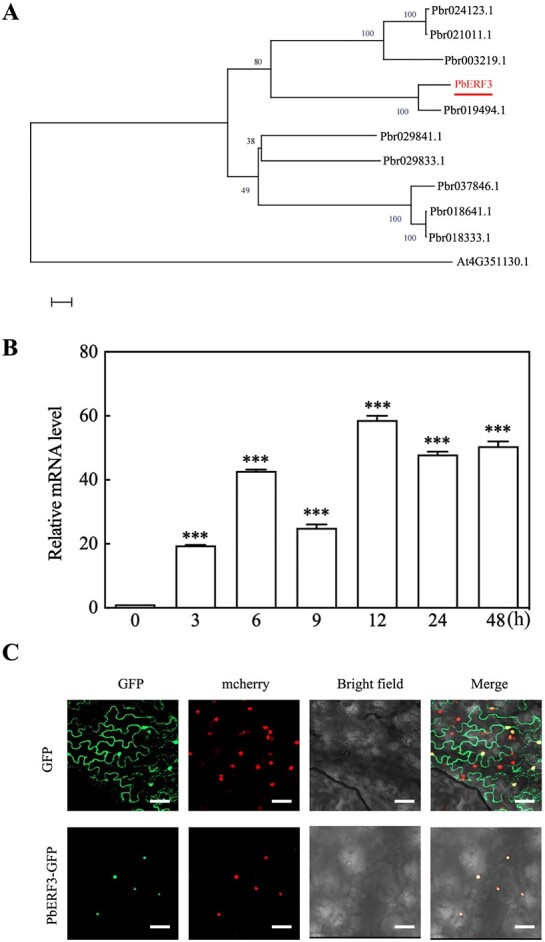
Identification and characterization of drought-responsive PbERF3. (A) The protein sequences utilized for the construction of the phylogenetic tree by MEGA7. (B) The relative expression of PbERF3 with drought is shown. (C) Epidermal cells of tobacco leaves expressing the fusion plasmid (*35S-PbERF3*) transiently. The nucleus is stained by DAPI stain.

PbERF3 had a molecular weight of 43.122 kDa and an isoelectric point of 7.96 amino acids. It was further discovered that *PbERF3* and *ERF3* in *Arabidopsis* shared a close genetic relationship (Fig. 1A). After *P. betulaefolia* seedlings were subjected to dehydration, quantitative reverse transcription polymerase chain reaction (qRT-PCR) revealed an increase in *PbERF3* expression (Fig. 1B). This suggests that *PbERF3* is involved in the response to dehydration stress. To provide additional evidence of the functional localization of PbERF3, a *PbERF3-GFP* vector was generated and expressed in tobacco plants. The *PbERF3-GFP*was located within the nucleus (Fig. 1C).

### PbERF3 positively regulates the response to drought stress in pears

**Figure 2:**
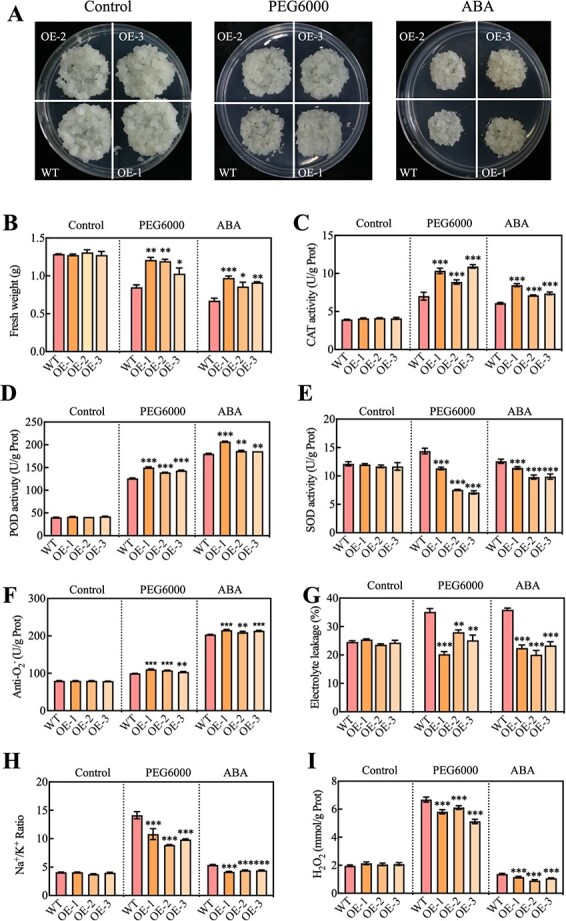
The PbERF3 overexpression callus exhibited reduced stress-induced damage. (A) Images displaying callus formation in the wild-type “Orin” callus and three transgenic callus lines (OE-1, OE-2, and OE-3) with overexpression of *PbERF3*. The photos were captured during the cultivation of the callus on a medium with PEG6000 and ABA for 7 days at 24°C. (B) Fresh pear callus weights comparable to those illustrated in (A) were taken for experimentation. The CAT activity (C), POD activity (D), SOD activity (E), Anti-O_2_^−^ (F), EL (G), Na^+^/K^+^ ratio (H), and H_2_O_2_ content (I) were detected.

**Figure 3:**
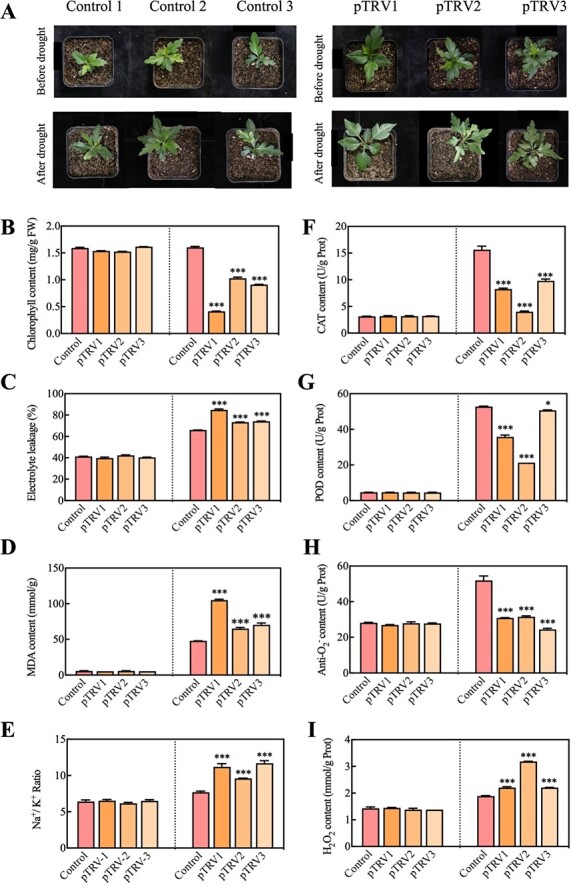
Virus-induced gene silencing of PbERF3 led to increased sensitivity to drought in *P. betulaefolia*. (A) Phenotypes of plants with silenced PbERF3 under drought stress. Chlorophyll content (B), EL (C), MDA content (D), Na^+^/K^+^ ratio (E), CAT activity (F), POD activity (G), Anti-O_2_^−^ content (H), and H_2_O_2_ content (I) of control and PbERF3-pTRV silenced plants (pTRV1, pTRV2, and pTRV3) after drought stress.

The OE of PbERF3 in callus (OE-1, OE-2, and OE-3) of pears (*Pyrus communis*) was detected by PCR and western blot (Fig. S1A, B), and its silencing in *P. betulaefolia* seeds (PbERF3-pTRV) was performed to determine the biological role of PbERF3 in abiotic stress. In stressful circumstances brought on by 10% PEG6000 and 5 μM ABA, the OE lines showed a higher level of the relative area (RA) (Fig. 2A), fresh weight (Fig. 2B), catalase (CAT) activity (Fig. 2C), peroxidase (POD) activity (Fig. 2D), superoxide dismutase (SOD) activity (Fig. 2E), and Anti-O_2_^−^ (Fig. 2F) than the wild type (WT), but lower electrolyte leakage (EL) (Fig. 2G) than WT. Interestingly, the Na^+^/K^+^ ratio (Fig. 2H) and H_2_O_2_ content (Fig. 2I) were lower than the WT group under PEG6000 and ABA stress. When subjected to PEG6000 stress, the qRT-PCR analysis showed that the expression levels of *NCED4*, *AA08OX*, *PYL2*, and *PP2C24* were higher in the OE lines compared to the WT (Fig. S1C). The results indicate that PbERF3 relies on the ABA signaling system to respond to osmotic and oxidative stressors. *P. betulaefolia* seedlings subjected to drought stress were found to be more susceptible to adverse conditions compared to the control seedlings, as evidenced by a reduction in *PbERF3* gene expression ranging from 60% to 50% in silencing lines (Fig. 3A; Fig. S2A). Under drought treatment, the seedlings treated with virus-induced gene silencing (VIGS) exhibited decreased chlorophyll content (Fig. 3B), higher EL (Fig. 3C), increased malondialdehyde (MDA) content (Fig. 3D), and a higher Na^+^/K^+^ ratio (Fig. 3E) compared to the control seedlings. The VIGS-treated seedings had lower CAT activity (Fig. 3F), POD activity (Fig. 3G), and Anti-O_2_^−^ (Fig. 3H), but higher H_2_O_2_ content (Fig. 3I) than the control group. In conclusion, PbERF3 responded positively to drought stress.

### PbERF3 enhances drought tolerance in transgenic *Arabidopsis*

**Figure 4:**
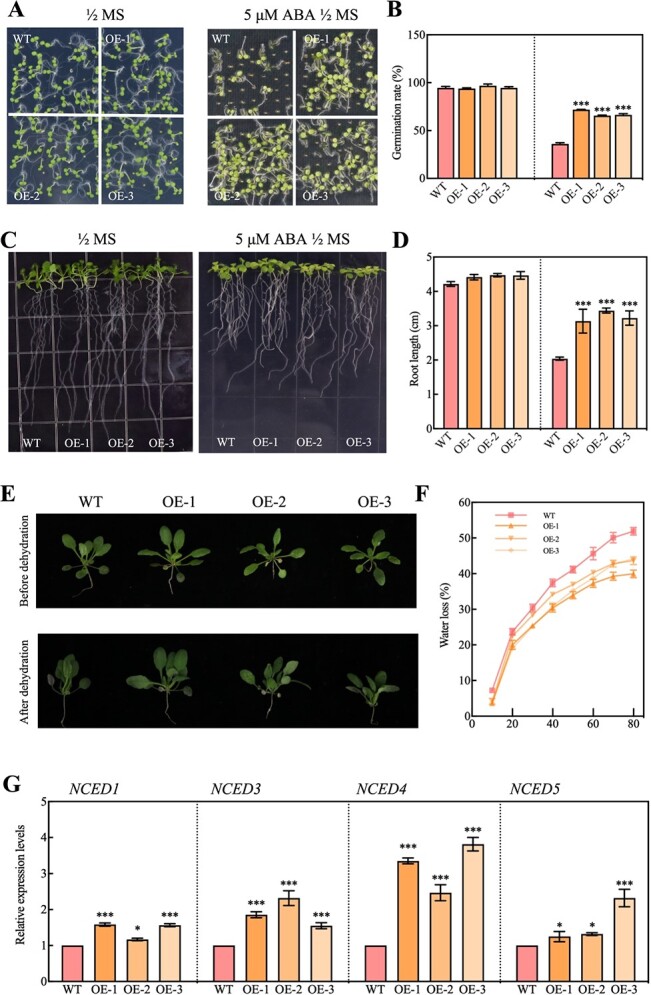
PbERF3 relies on the ABA signaling pathway. (A) Images of germinated WT and PbERF3 (OE-1, OE-2, and OE-3) seedlings on 1/2 MS media 10 days post-germination. (B) (B) shows a quantitative analysis of the percentage of seedlings exhibiting the cotyledon development. (C) Five-day-old WT and PbERF3 overexpression plants (OE-1, OE-3, and OE-3) were moved to a medium with 5 μM ABA. (D) Following stress as shown in (C), relative primary root elongation was observed. (E) The WT and PbERF3 transgenic plants were dehydrated. (F) The dehydration rates were detected in (F). (G) The expression of *NCEDs* was detected under dehydration.

**Figure 5:**
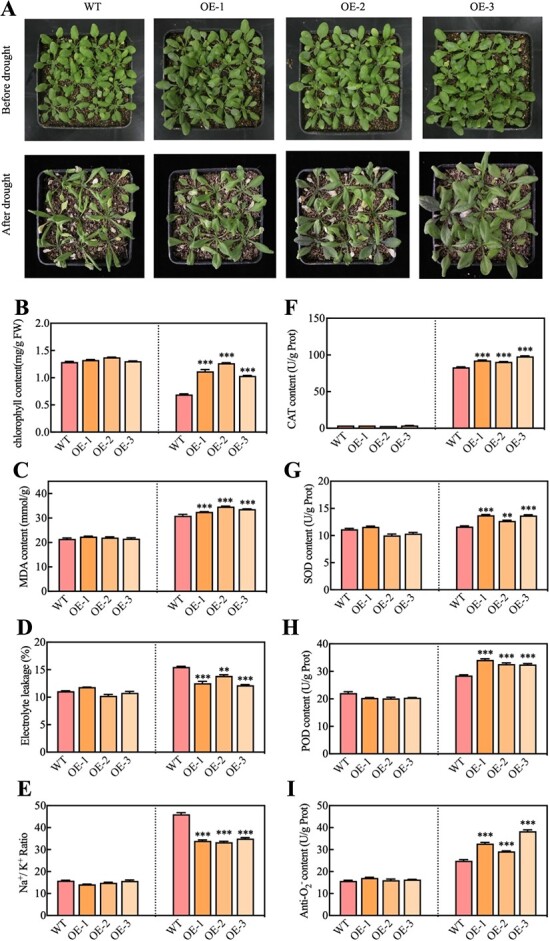
Transgenic PbERF3 lines showed increased drought tolerance. (A) The 35-day-old WT and PbERF3 transgenic plants were subjected to a 9-day drought treatment. (B–I) chlorophyll content (B), MDA content (C), EL (D), Na^+^/K^+^ ratio (E), CAT activity (F), SOD activity(G), POD activity (H), and Anti-O_2_^−^ content (I) were detected before and after drought stress.

A gain-of-function approach was employed to analyze the functionality of Arabidopsis. PCR and western blot analysis were utilized to identify the transgenic OE lines (OE-1, OE-2, and OE-3; Fig. S3A, B). The application of a 5μM concentration of ABA had a considerable inhibitory effect on the growth of green cotyledons in WT *Arabidopsis*. However, this reduction in inhabitation was partially reversed by overexpressing PbERF3, as seen in Fig. 4A and B. Under 5μM ABA treatment, the OE lines promoted root growth compared to WT plants (Fig. 4C, D). After experiencing dehydration stress, the OE lines showed a reduction in wilting compared to the WT lines (Fig. 4E, F). Under PEG6000 treatment, the OE lines promoted root growth compared to the WT plant (Fig. S4A, B), and the expression level of the key enzyme NCEDs (*NCED1*, *NCED3*, *NCED4*, *NCED5*) in ABA biosynthesis was upregulated under dehydration treatment (Fig. 4G). The results demonstrated that PbERF3 *Arabidopsis* has the potential to enhance drought resistance by modulating the ABA signal pathway. After experiencing drought stress, the wilting symptom of the PbERF3 plant was less apparent in comparison to the WT (Fig. 5A, Fig. S4C). In addition, the PbERF3 plant exhibited elevated levels of chlorophyll and MDA compared to the WT group, as shown in Fig. 5B and C. However, it had reduced levels of EL and Na^+^/K^+^ ratio compared to the WT group (Fig. 5D, E). The CAT, SOD, POD activity, and Anti-O_2_^−^ content were higher than those of the WT (Fig. 5F–I). These results suggested that PbERF3 beneficially controlled drought stress.

### PbERF3 stimulates the transcription of *PbPIP1;4*

**Figure 6:**
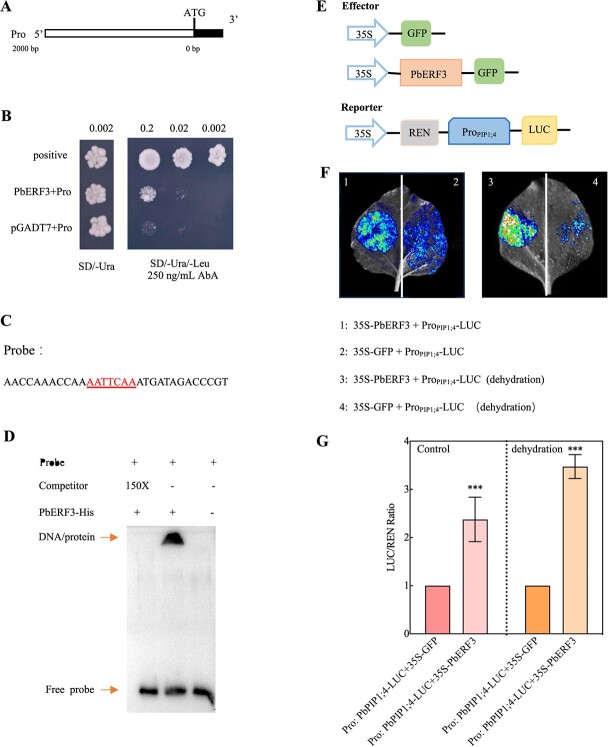
PbERF3 activate *PbPIP1;4.* (A–B) The experiment was conducted on an SD medium containing aureobasidin A (AbA). The *pGADT7-p53* and *pABAi-p53* constructions were used as the positive controls, while the *pGADT7* and Pro (*PbPIP1;4-pAbAi*) constructs were used as the negative controls. (C) The probe sequence includes a potential PbERF3 binding motif, AATTCAA (highlighted). (D) The His-PbERF3 protein interacted with the *cis*-element, as shown by the EMSA experiment. The unlabeled fragment served as a competitor; − indicates absence and + indicates presence. (E–G) A visual representation showing an *N. benthamiana* leaf 48 h after being infused with the PbPIP1;4 promoter. The fluorescence imaging is shown in (F). Different effector and reporter constructs utilized on tobacco leaves are illustrated schematically. The LUC/REN ratios were obtained after the leaves were co-infiltrated with certain combinations of constructs (G).

To get a more comprehensive understanding of the molecular mechanism behind its reaction to drought stress, the Y1H test validated the correlation between the PbREF3 and *PbPIP1;4* promoter. The promoter region of *PbPIP1;4* was included in the *pABAi* vector (Fig. 6A). Yeast cells expressing the *PbPIP1;4* promoter grew normally with the introduction of 250 ng/mL (AbA). However, the growth of yeast cells expressing the *pABAi* vector was inhibited (Fig. 6B). Utilizing an electrophoretic mobility shift assay (EMSA), the binding of PbERF3 to the *PbPIP1;4* promoter was further validated. The binding signal was detectable when the probe and PbERF3-His protein were added, but it was eliminated when the competitor was introduced (Fig. 6C, D). These results suggest that PbERF3 interacts with *PbPIP1;4* promoter.

An assay for transient expression in *N. benthamiana* was employed to verify that PbERF3 could activate *PbPIP1;4*. Next, it was investigated whether PbERF3 promotes *PbPIP1;4* gene transcription *in vivo* using a transient expression mechanism in *N. benthamiana* leaves, for which the promoter of *PbPIP1;4* was ligated to the *pCAMBIA 1381Z-LUC* vector. Upon transient expression of the *PbPIP1;4* promoter construct in the leaves, a significant increase in luciferase (LUC) activity was detected compared to the control group (Fig. 6F). In the presence of the effector and reporter constructs, the LUC/renilla (REN) ratio was higher than in the control, particularly under conditions of dehydration (Fig. 6G). These results suggest that PbERF3 directly stimulates the activation of *PbPIP1;4* and that PbEFR3 is capable of transmitting signals through the activation of *PbPIP1;4*.

### PbERF3 interacts with PbHsfC1a

**Figure 7:**
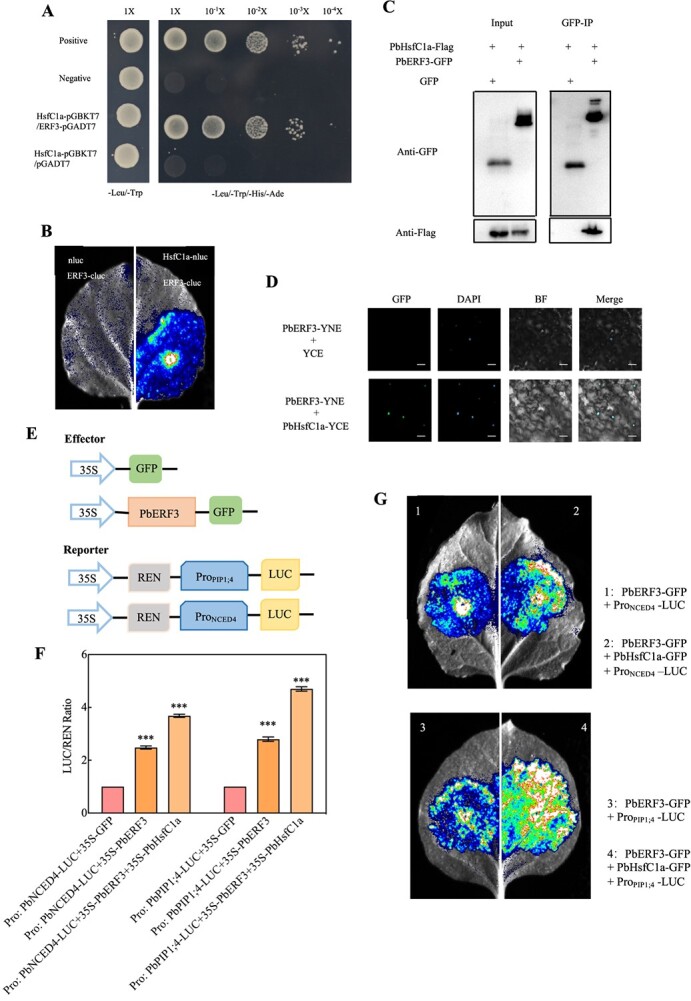
PbERF3 interacted with PbHsfC1a activates *PbPIP1;4* and *PbNCED4*. (A) AH109 yeast cells were grown on a selection medium. PbERF3 (bait) and PbHsfC1a (prey) were introduced accordingly. (B) The *PbHsfC1a* was fused to the N terminus of the firefly LUC protein, leading to the formation of a new protein called PbHsfC1a-nluc. The *PbERF3* was fused to the C terminus, resulting in the formation of PbERF3-cluc. The co-infiltration of *nluc*/*PbERF3-cluc* was employed as a negative control. Fluorescence was detected as a result of the simultaneous introduction of *PbHsfC1a-nluc* and *PbERF3-cluc*. (C) *GFP/PbHsfC1a-Flag* and *PbERF3-GFP/PbHsfC1a-Flag* constructs were transfected into *N. benthamiana* leaves, and protein extracts were immunoprecipitated (IP) using GFP beads detected with antibodies. (D) The PbERF3 protein was combined with *YFP*, resulting in the fusion protein PbERF3-YNE. Similarly, the PbHsfC1a protein was fused with *YCE*, resulting in the fusion protein PbHsfC1a-YCE. This (*YCE*/*PbHsfC1a-YNE*) was employed as a negative control. (E–G) PbERF3 and PbHsfC1a transactivated *PbPIP1;4* and *PbNCED4*; the representative images are displayed (E). Transactivation of *PbPIP1;4* and *PbNCED4* promoter by PbERF3 and PbHsfC1a was analyzed, LUC/REN ratio was detected as shown in (F). The fluorescence images are represented in (G).

To explore the molecular mechanism of PbERF3, a Y2H screening was conducted using a *P. betulaefolia* cDNA library, with PbERF3 serving as the bait. Many TFs, including *PbHsfC1a* (Pbr014107.1), were detected. Through the process of co-transforming the *PbERF3-pGADT7* and *PbHsfC1a-pGBKT7* constructions into yeast, the association between PbERF3 and PbHsfC1a using a yeast 2-hybrid (Y2H) test was successfully identified. This assay confirmed the interaction between PbERF3 and PbHsfC1a within the yeast cells (Fig. 7A). The split-LUC test (Fig. 7B) showed that only the constructs with fused PbERF3 and PbHsfC1a generated a stronger signal in *N. benthamiana* leaves, indicating that the proteins interacted in plants. Tobacco leaves were transiently co-transfected with different combinations of constructs for the co-immunoprecipitation (Co-IP) assay, such as *PbHsfC1a-Flag/PbERF3-GFP* and *PbHsfC1a/GFP*, as identified using immunoblot analysis. The results of the assay showed that PbERF3 interacted with PbHsfC1a (Fig. 7C). For the bimolecular fluorescence complementation (BiFC) assay, the fluorescence of only those tobacco leaves expressing both PbERF3-YNE and PbHsfC1a-YCE was detected, compared to the control group (Fig. 7D). Collectively, these results suggest that PbERF3 and PbHsfC1a engage in an interaction both *in vitro* and *in vivo*.

### PbERF3-PbHsfC1a function together as transcriptional activators for *PbPIP1;4* and *PbNCED4*

PbHsfC1a has demonstrated the ability to bind to the promoters of *PbPIP1;4* and *PbNCED4*, hence regulating H_2_O_2_ transport and ABA biosynthesis [30]. The transient expression system was utilized to investigate the activation of the *PbNCED4* by PbERF3. The LUC/REN ratio was not greater than that of control (Fig. S5A, B). To further explore the molecular mechanism of the interaction between PbERF3 and PbHsfC1a, a dual luciferase experiment was used to confirm that PbERF3 and PbHsfC1a could jointly activate *PbPIP1;4* and *PbNCED4*. *GFP/PbPIP1;4-0800*, *PbERF3-GFP/PbPIP1;4-0800*, *PbHsfC1a-GFP*/*PbERF3-GFP/PbPIP1;4-0800*, *PbERF3-GFP/PbNCED4-0800*, *PbHsfC1a-GFP*/*PbERF3-GFP/PbNCED4-0800*, or *GFP/PbNCED4-0800* was co-transformed into tobacco leaves. The LUC/REN ratio was observed to be higher than that of the control (Fig. 7E, F), demonstrating that PbERF3 interacted with PbHsfC1a and coordinately activated *PbPIP1;4* and *PbNCED4*. Moreover, an investigation was conducted to determine if PbERF3 and PbHsfC1a cooperatively contributed to the increased expression of *PbPIP1;4* and *PbNCED4*. The promoters of *PbPIP1;4* and *PbNCED4* were combined with the *pCAMBIA 1381Z-LUC* vector. The leaves that transiently expressed the PbERF3 and PbHsfC1a construct exhibited a higher level of LUC activity compared to the control group (Fig. 7G). The above results indicate that PbERF3 interacted with PbHsfC1a to activate *PbPIP1;4* and *PbNCED4* coordinately.

## Discussion

In plant growth and development, ERF TFs are essential regulators [11], involved in cellular differentiation, nutrient transport [31], and hormone signaling [32], etc., contributing to overall plant growth and yield [33]. ROS-mediated redox regulation influences both plant development and stress response [34, 35]. However, the mechanism by which plants perceive external stimuli and generate internal responses remains unidentified. In this study, it has been shown that the PbERF3-PbHsfC1a model increases the expression of *PbPIP1;4* and *PbNCED4* to protect the plant against drought-mediated damage. To enhance crop resilience and resistance to drought and to mitigate the adverse impacts of drought on agricultural and natural plant ecosystems, it is crucial to understand the intricate responses of plants to drought-induced stress.

ERFs are TFs that are unique to plants. Many of them facilitate the transcription of genes that are dependent on ethylene [36]. ERF proteins are capable of binding to DNA *cis*-acting elements, including AA(T)TTCAA motifs, DRE (CCGAC), and GCC box (AGCCGCC), *via* their conserved ERF domain [37]. These motifs have been identified in the promoter region of various ethylene-responsive signaling or biosynthesis genes [38]. In this study, it was found that PbERF3 was able to bind to *PbPIP1;4* promoters *via* AATTCAA element (Fig. 6). PbERF3 interacted with *PbPIP1;4* to enhance H_2_O_2_ transport. Furthermore, PbERF3 formed an interaction with PbHsfC1a to increase the transcription of *PbNCED4*, which is involved in the biosynthesis of ABA (Fig. 7). Various studies have indicated that ABA has an impact on the synthesis of ethylene by presumably regulating the amounts of 1-aminocyclopropane-1-carboxylate (ACC) synthase and ACC oxidase [39]. The current study added the mechanism of action of ABA and H_2_O_2_ signaling molecules in the ethylene signaling pathway. This enhances the intricate function of secondary metabolites in the signaling cascade and offers vital insights for further investigating the complex regulatory network, including ABA, H_2_O_2_, and ethylene.

ROS serves as a crucial indicator for assessing the extent of oxidative damage in plants . Antioxidant enzymes are enzymes that remove ROS and have a crucial function in the enzyme system. They can decrease the build-up of ROS, which helps to combat oxidative stress [34]. In this investigation, it was found that the PbERF3 lines that were overexpressed had a lower accumulation of ROS compared to the WT (Figs. 2 and 5). However, the silenced plants gathered an excessive quantity of ROS when subjected to drought stress (Fig. 3). Studies have shown that PbHsfC1a interacted with *PbPIP1;4* promoter region and enhances *PbPIP1;4* expression. PbPIP1;4 can transport H_2_O_2_ and stimulate the downstream cascade signaling pathways [30]. The function of PbPIP1;4 in transporting H_2_O_2_ is of great significance in the response to drought stress [30]. The present results suggest that PbERF3 can stimulate *PbPIP1;4*, leading to an increase in the activities of plant antioxidant enzymes. This helps in the elimination of ROS that are generated during drought stress, as shown in Fig. 6. Plants experience a range of biotic and abiotic stresses, leading to rapid alterations in the production and elimination of ROS, such as H_2_O_2_ [5]. The discovery revealed a novel molecular mechanism that, in response to drought stress, rebuilds redox homeostasis *via PbPIP1;4*.

**Figure 8:**
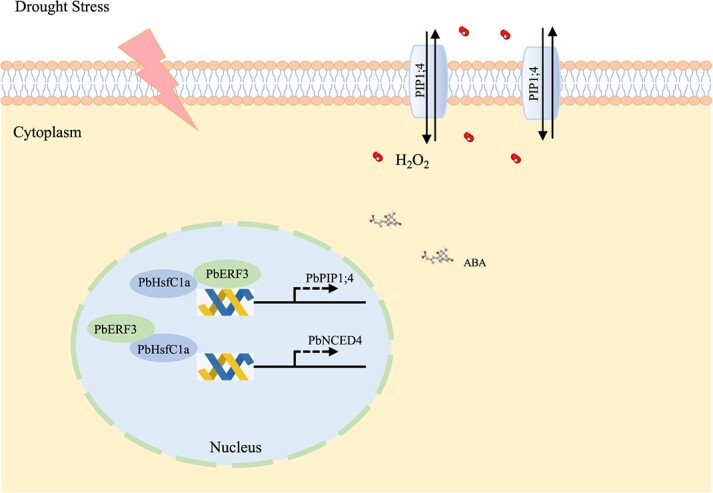
A model of PbERF3 interacting with PbHsfC1a activates *PbNCED4* and *PbPIP1;4* linked ABA and H_2_O_2_ signal to resistance to drought stress.

Although ABA has a variety of roles in plant growth and development, its primary role is to maintain plant water balance and osmotic stress tolerance [9]. The precise mechanism by which osmotic stress sensing and the activation of ABA-producing genes interact remains uncertain [36]. In apple, ABA is a major element involved in apple production, and important in seed germination and early seeding development [33]. Therefore, it is crucial to explore the mechanism of endogenous synthesis of ABA in *Rosaceae*. PbHsfC1a directly binds to *PbPIP1;4* and *PbNCED4* promoter and improves drought tolerance together with H_2_O_2_ downstream cascading signals and ABA signal pathway [30]. In this study, under ABA treatment, the PbERF3 OE plant showed a higher germination rate compared to the WT (Fig. 4). PbERF3 binds directly to the promoter of *PbPIP1;4* in response to H_2_O_2_ signaling. PbERF3 and PbHsfC1a form a heterodimeric oligomer that binds to the *PbNCED4* promoter, hence enhancing ABA production (Fig. 6). The regulation of the cell cycle and other cellular activities by ABA may potentially be responsible for the reduced height observed in *aba* mutants [40]. ABA is crucial for the growth and development of pear trees. Conducting more research on the impact of ABA on fruit tree output is particularly important for addressing the overall challenge faced by the fruit tree business industry.

Here, a working model for the function of PbERF3 under drought stress has been presented based on the findings of this investigation (Fig. 8). PbERF3 is activated in response to drought and forms an interaction with PbHafC1a. The heterologous oligomer PbERF3-PbHafC1a controls the expression of genes related to ABA biosynthesis and H_2_O_2_ transport by binding to the promoters of *PbPIP1;4* and *PbNCED4*. This study revealed a novel method by which PbERF3 conferred drought tolerance to the pear plant, providing vital information for developing drought-resistant crops.

## Experimental procedures

### Plant materials and stress treatment

The expression pattern of PbERF3 was analyzed under drought stress circumstances by cultivating 40-day-old seedlings of *P. betulaefolia* leaves in the conservatory of the National Center of Pear Engineering Technology Research, Nanjing Agricultural University. The 45-day-old seedlings were treated for dehydration by placing them on a dry filter paper in an ambient condition for 0, 1, 3, 6, 9, 12, 24, 36, and 48 h. At specific intervals, three seedlings were harvested at random to collect samples. The expression of the *PbERF3* genes generated the *35S-PbERF3* lines. The T3 transgenic lines that had been chosen were used for the subsequent studies. The previously disclosed methodologies for bioinformatics analysis were used as a reference [30].

### Vector construction and plant transformation

The recombinant vector was generated through the cloning process of the CDS *PbERF3* into the *pCAMBIA-1300-GFP* vector. The vectors were employed to modify *Arabidopsis* and pear callus by transferring them into *Agrobacterium* GV3101.

### The localization of PbERF3

A few adjustments were made following the methodology reported in a previous study [30]., 2019). The *PbERF3* was inserted into the *pCAMBIA-1300-GFP* vector and fused in-frame with the N terminus of Green Fluorescent Protein (GFP). The location of the nucleus was determined using 4',6-diamidino-2-phenylindole (DAPI) labeling.

### Sodium Dodecyl Sulphate -Polyacrylamide Gel Electrophoresis (SDS-PAGE) and immunoblotting

The protein extracts were separated using a 10% SDS-PAGE method. Following electrophoresis, the protein was transferred onto a polyvinylidene difluoride membrane, and the electrophoresis was run for 50 min at 100 V. The membrane was blocked using a 5% blocking solution containing skimmed milk, and it was allowed to incubate at room temperature for 2 h under gentle shaking. Immunoblot screening was conducted using specific antibodies.

### Green open cotyledon assays

Stratified seeds were allowed to germinate on 1/2 Murashige & Skoog (MS) medium, either with or without ABA, at the designated concentrations. Photographs were obtained to represent the samples, and several characteristics, such as the appearance of green cotyledons and the length of the primary root, were measured [30]. All manipulations were performed at 25 ± 1°C.

### Expression and purification of recombination

The full-length coding sequence of PbERF3 was inserted into the *PET28α* vector for His-tagged fusion according to Table S1. All methods strictly followed the instructions provided by the manufacturer. The His-PbERF3 proteins were isolated by purification utilizing an Ni-NTA prepacked gravity column.

### Y1H assay

Following the manufacturer’s guidelines, the Y1H assay was conducted (Clontech). The promoter region was inserted into the *pABAi* vector (Table S1). The recombination constructs, *pGADT7-p53*, and *pABAI-p53* served as a positive control in the Y1H bait strain and were cultivated on a dropout/-Leu/-Ura plate with aureobasidin A(AbA).

### Electrophoretic mobility shift assay

The probe was selected to be the promoter region having AATTCAA. To attach biotin labels to the WT oligonucleotides for EMSA probes, the instructions provided by the manufacturer were followed, and an EMSA Probe Biotin Labeling Kit (Beyotime, Shanghai, China) was utilized [30].

### LUC assay

The promoters were included in the *pGreen 0800 LUC* vector. Following incubation, the cells were harvested and then resuspended in an infiltration buffer. This solution consisted of 50 mM MgCl_2_, 200 mM MES, and acetosyringone combined at 600 nm. The *Agrobacterium* strains carrying the specified constructs were added to the buffer at a final optical density of 0.4. Plants were subjected to 48 h of darkness at a temperature of 25°C. Activity measurements of the LUCs for firefly (*Photinus pyralis*) and Renilla (*Renilla reniformis*) were taken sequentially from a single sample. The transcriptional efficiency was assessed by calculating the ratio of firefly to Renilla LUC activity [35].

### Co-IP assay

The *PbERF3-GFP* constructs were generated by inserting the whole protein into the 1300-GFP vector to prepare for the Co-IP assay. The *N. benthamiana* leaves displayed a transitory expression of the constructions. The proteins from tobacco leaves were extracted using the Co-IP buffer, as described previously [35]. The immunoprecipitated proteins were examined using antibodies.

### Dual luciferase complementation assay

*N. benthamiana* leaves were transfused with constructs encoding *PbERF3-cluc/PbHsfC1a-nluc* and *PbERF3-cluc/nluc*. The constructions demonstrated expression for 48 h, and the signals were measured using chemiluminescence imaging.

### BiFC assay

The BiFC analysis employed *pSPYNE* and *pSPYCE* as vectors. The constructs *PbERF3-YNE/PbHsfC1a-YCE* or *-YNE/PbHsfC1a-YCE* were inserted into *N. benthamiana* leaves and allowed to express for 48 h. The signals were detected using chemiluminescence imaging.

### Statistical analyses

Each sample was analyzed using three independent experimental replicates, with the results presented as mean ± standard error (*n* = 3). Analysis of variance was used to examine the statistical differences determined by Duncan’s multiple range test at significance levels of *P* <0 .05 (*), *P* <0 .01 (**), and *P* <0 .001 (***).

#### ACCESSION NUMBERS

Accession numbers of the genes mentioned in this study are as follows: PbERF3 (Pbr030451.1), PbPIP1;4 (Pbr009491.1), and PbNCED4 (Pbr034869.1).

## Acknowledgments

This work has been supported by the National Key Research and Development Program of China (2019YFD1000102), the Key Research and Development Program of Jiangsu Province (BE2023328), the National Science Foundation of China (32072538), the Jiangsu Agriculture Science and Technology Innovation Fund (CX(22)3046), and Postgraduate Research and Practice Innovation Program of Jiangsu (KYCX23_0795).

## Author Contributions

X.S.H., F.Z., and Z.J.P. designed the project and experiments. F.Z. and Z.J.P. performed most of the experiments. C.Y.H., H.Z.D., L.K.L., Q.H.Q., and K.K.Z. analyzed the data. S.L.Z. provided the research platform. X.S.H., S.T.T., and J.Y.W. directed the manuscript. All authors have read, evaluated, and endorsed the final manuscript.

## Data Availability

All relevant data can be found within the manuscript and its supporting materials.

## Conflict of interest statement

All authors disclosed no relevant relationships.

## Supplementary data

Supplementary data is available at *Horticulture Research* online.

## Supplementary Material

Web_Material_uhae090
