# Supplementary figures and images for: Pyrus betulaefolia ERF3 interacts with HsfC1a to coordinately regulate aquaporin PIP1;4 and NCED4 for drought tolerance
Source: Hortic Res. 2024 Mar 30;11(5):uhae090. doi: 10.1093/hr/uhae090 (PMC11116902; doi:10.1093/hr/uhae090)

Figure S1

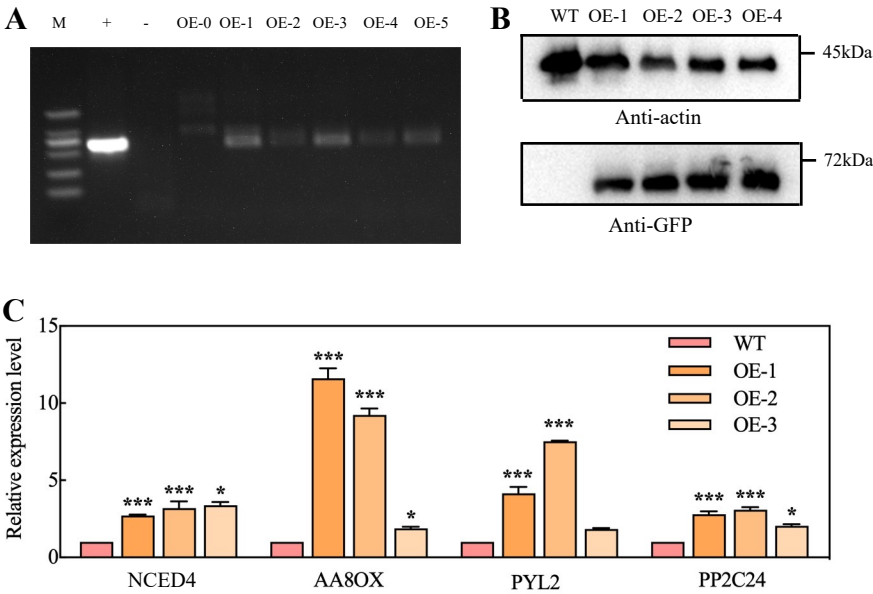

Figure S2

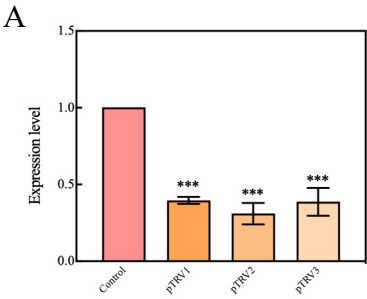

Figure S3

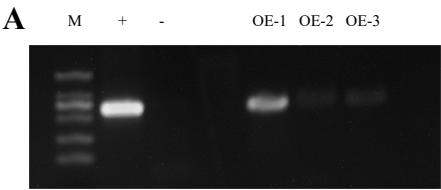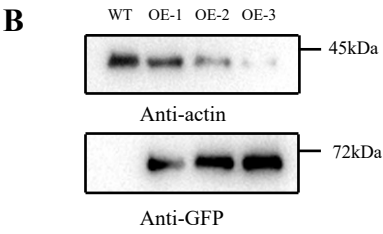

Figure S4

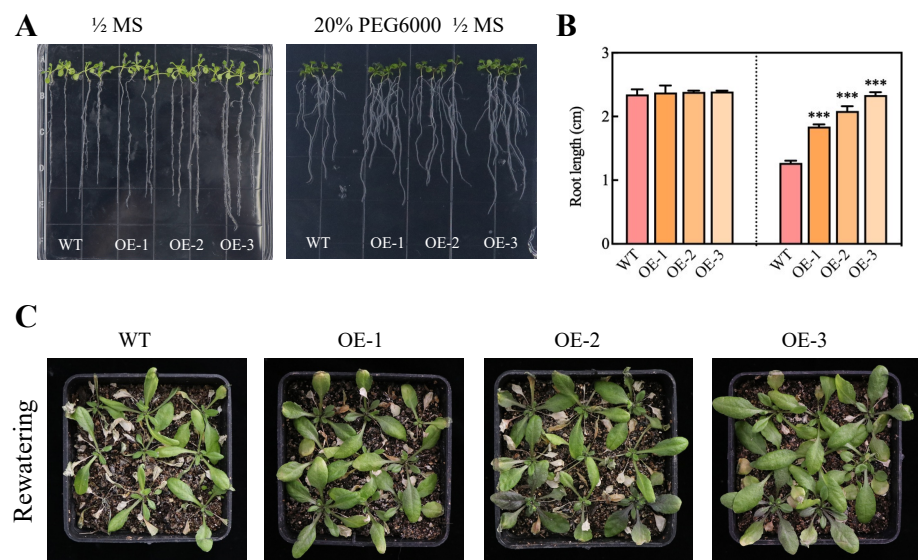

Figure S5

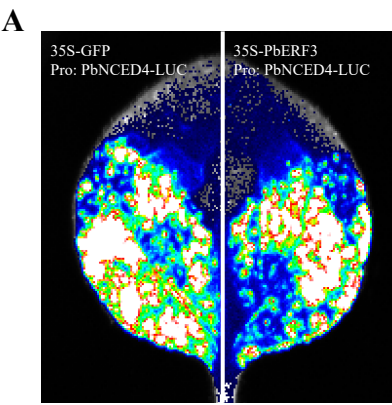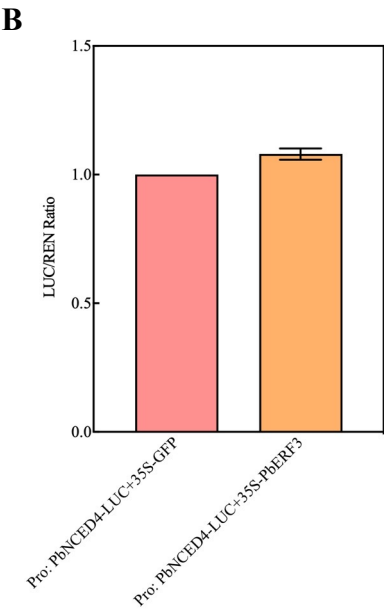

Supplement: Web_Material_uhae090 [file web_material_uhae090.zip › Supplemental Figures.pdf]
